# Retrospective Cohort Study of Intraoperative Administration of Sustained-Release 5-Fluorouracil Implants in Advanced Gastric Cancer Patients

**DOI:** 10.3389/fphar.2021.659258

**Published:** 2021-04-13

**Authors:** Jie Ge, Ting Liu, Tianxiang Lei, Xuan Li, Kun Song, Samim Azizi, Heli Liu, Mimi Tang

**Affiliations:** ^1^ Department of Gastrointestinal Surgery, Xiangya Hospital, Central South University, Changsha, China; ^2^ Department of Pharmacy, Xiangya Hospital, Central South University, Changsha, China; ^3^ Department of Cardiothoracic and Vascular Surgery, Xiangya Hospital, Central South University, Changsha, China

**Keywords:** sustained-release 5-fluorouracil, Gastric cancer, safety, Long-term effect, postoperative chemotherapy

## Abstract

**Background:** 5-fluorouracil (5-FU) is basically used in the field of postoperative chemotherapy of gastric cancer (GC), the goal of this study was to evaluate improvement of long-term survival rate among GC patients after the 5-FU implants treatment.

**Methods:** The study included 145 patients with gastric cancer who received postoperative chemotherapy with 5-FU implants and had complete follow-up information. According to the sex, age and clinical stage of 5-FU implants group, 74 patients were matched as the control group at the same time. In the study, we compared the 5-year overall survival rate with progression-free survival rate in the two groups, and the drug safety for both groups during the treatment was also compared.

**Results:** The median follow-up time was 85 months (range 60–116 months). 31 patients (21.38%) died of tumor recurrence in 5-FU implants group and 21 (28.38%) in control group. In the control group, metastatic lesions were found in the small intestine, left adrenal gland and peritoneum in three patients. The 5-year progression-free survival (PFS) rate was 79.71% in 5-FU group and 67.12% in control (*p* = 0.0045). The 5-year overall survival (OS) rate was 77.68% in 5-FU implants group and 64.87% in control (*p* = 0.0159). Both the 5-years OS and PFS rates in 5-FU group were better than control group without significant side effect.

**Conclusions:** 5-FU implants may improve 5-years OS and PFS rates after surgery in gastric cancer patients, while good safety profile suggests it could be reliable.

## Introduction

Gastric cancer (GC) is one of the three major causes of death among cancer patients, which seriously threatens human health (Petryszyn et al., 2020). According to the global cancer statistics in 2018, there were more than 1 million new cases of GC worldwide, resulting in over 782,000 deaths (Bray et al., 2018). Regional differences play a crucial role in the incidence of GC.

A study showed that nearly half of the global cases in 2017 occurred in China (GBD 2017 Stomach Cancer Collaborators, 2020). The combination of treatments including surgical resection, radiotherapy and chemotherapy has been recommended as the standard of care for GC (Tan, 2019; Farhat et al., 2010). Although GC patients may be cured by surgical resection, about 60% of patients still relapse after surgery (Deng et al., 2011; Liu et al., 2016). Since it severely affects the survival rate of GC with an incidence of greater than 50% (Deng et al., 2011) and according to the data of our department, the peritoneal metastasis rate is as high as 86% in patients with stage IV gastric cancer (unpublished data), the prevention of peritoneal metastases after radical surgery has become an increasing concern.

Although many methods have been developed to conquer the peritoneal metastasis, such as hyperthermic intraperitoneal chemotherapy (HIPEC) (Yarema et al., 2019), pressurized intraperitoneal erosol chemotherapy (PIPAC) (Sgarbura et al., 2019), the outcome of GC with peritoneal metastasis is still unsatisfied. Intraperitoneal (IP) infusion of chemotherapy drugs offers an attempt to expose cancer cells to anti-cancer drugs at high drug concentrations with minimal systemic toxic effects (Shinkai et al., 2018), but implantation of the port may cause some complications. Yang et al. reported that 22.9% patients experienced port complications, including subcutaneous liquid accumulation, infection, port rotation, inflow obstruction, and even subcutaneous metastasis (Yang et al., 2020). Facing such urgent situation, there is a great need for carrying out some simpler, safer methods with high effective to prevent peritoneal metastasis and prolong the survival of GC patients.

Nowadays, 5-fluorouracil (5-FU) is the most basic chemotherapeutic agent for GC. Combination of 5-FU and multiple chemotherapeutic drugs can increase the efficacy rate and improve the quality of life with GC patients (Jung et al., 2009). For patients after radical resection, who accepted 5-FU and leucovorin adjuvant chemotherapy, the five-year survival rate and recurrence-free survival rate reported to be 60 and 57% respectively (Park et al., 2003). The Japan Clinical Oncology Group Study (JCOG) 9,205 showed that the overall survival (OS) rate of the 5-FU regimen alone and cisplatin combination were similar (28 vs. 29%) in the unresectable advanced GC patients (Ohtsu et al., 2003). Consequently, 5-FU is listed as a recommended drug, which included European Society for Medical Oncology (ESMO), National Comprehensive *Cancer* Network (NCCN), Chinese Society of Clinical Oncology (CSCO), and Japanese gastric cancer treatment guidelines (Ajani et al., 2016; Smyth et al., 2016; Wang et al., 2019; Japanese Gastric Cancer Association, 2020).

5-FU has a short half-life and is quickly eliminated after entering the circulation *via* intravenous administration (Sun et al., 2017), whereas sustained-release 5-FU overcomes this disadvantage and is widely used in China. As one of the representatives of sustained-release preparations, the slow-release effect of 5-FU implants prolongs the action time of the drug in the body, improves safety, and maximizes drug utilization (Yuan et al., 2015).

In China, 5-FU is basically used for chemotherapy of GC, colorectal cancer, breast cancer, and pancreatic cancer. Presently, clinical studies on 5-FU sustained-release preparations have focused on colorectal cancer and liver cancer (Cheng et al., 2013; Yuan et al., 2015; Chen et al., 2016; Li et al., 2018), but rarely on GC. The aim of our study was designed to discuss and evaluate the possibilities of radical surgery combined with 5-FU implants in increasing long-term survival rate.

## Patients and Methods

### Patients

A total of *n* = 219 patients aged from 27 to 84 years with GC who had been treated in the Department of gastrointestinal surgery, Xiangya Hospital of Central South University between January 2011 and September 2015 were included in this study.

The inclusion criteria were: patients with 1) clinical stage II–IV accepted laparoscopic or open radical surgery; 2) gastric cancer confirmed by pathological examination; 3) a treatment without any other chemotherapy or radiotherapy before the operation; 4) data integrity.

The exclusion criteria were: patients with 1) palliative care; 2) gastric remnant cancer; 3) liver and kidney dysfunction; 4) any unsuitable condition for chemotherapy.

This study was approved by the ethics committee of Xiangya Hospital of Central South University. All included patients provided informed consent.

### Sustained-Release 5-FU Administration

**FIGURE 1:**
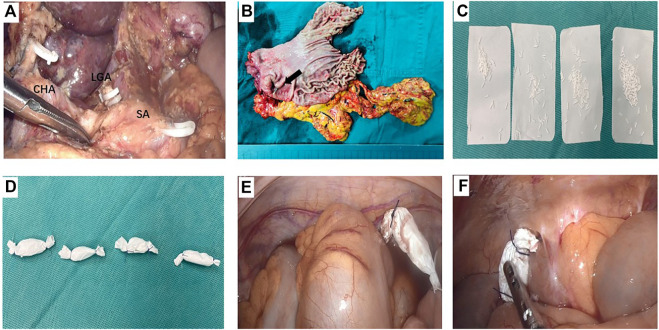
A representative case who received 5-fluorouracil (5-FU) implants during operation **(A)** The operation field of laparoscopic distant gastrectomy after D2 dissection. The three branches of the celiac trunk were exposed, i.e. splenic artery (SA), common hepatic artery (CHA), and the stump of left gastric artery (LGA) **(B)** The surgical specimen of gastric cancer located in the antrum (black arrow) **(C)** and **(D)** 800 mg 5-FU implants were divided into 4 parts and were packed by absorbable gauze **(E)** One pack of 5-FU implants was placed in the pelvic cavity **(F)** One pack of 5-FU implants was placed in the right paracolic sulci.

In 5-FU implants group, a total dose of 600–800 mg 5-FU implants (Sinofuan^®^, Wuhu Zhongren Pharmaceutical Co., Ltd. Anhui Province, China) was scattered near the tumor bed, pelvic cavity, paracolonic groove and subdiaphragma at the end of surgery. The dosage of drug was calculated by patients’ body surface area, ie, the dosage was 400 mg/m^2^. It’s worth noting that 5- FU implants were always positioned in standard sites to avoid the implants contact directly with the skeletoned vessels and anastomosizes. Meanwhile, where the surface of the small intestine, anastomosis and exposed vessels were carefully avoided (Figure 1).

### Safety Evaluation

The safety evaluation included three aspects: postoperative complications, hematological toxicity, and non-hematological toxicity. The complications recorded in the present study included pelvic infection, peritoneal infection, anastomotic leakage, pulmonary infection, incisional infection, and postoperative hemorrhage. In addition, length of hospital stay after operation, time to first exhaust and defecation were also recorded. The items examined in this clinical study were: 1) hematologic toxicity: red blood cell (RBC), white blood cell (WBC), platelet (PLT) counts; 2) hepatotoxicity: aspartate aminotransferase (AST), alanine aminotransferase (ALT); 3) renal toxicity: creatinine (Cr); 4) gastrointestinal toxicity: diarrhea, nausea, and vomiting; 5) fever. Toxicity was reported based on the National *Cancer* Institute common terminology criteria for adverse events (NCI-CTCAE v4.0) (Hara et al., 2011; Yuan et al., 2015).

### Statistical Analysis

Results from the clinical trial were conducted by the statistical computer software of SPSS version 19.0 (IBM SPSS Statistics 19.0) and presented as mean ± SD. One-way analysis of variance (ANOVA) and paired *t*-test were performed to investigate the hematologic toxicity within and between the groups to evaluate whether 5-FU implants would cause more adverse reactions. Complications and adverse effects were evaluated by group *t*-test. In addition, Bonferroni post hoc analysis was done for multiple pairwise comparisons. The five-year PFS and OS were estimated by the Kaplan-Meier method in GraphPad Prism 8.0, which were the primary endpoint and secondary endpoint respectively. To assess the importance of potential prognostic factors, univariate and multivariate analysis were performed using log-rank test and Cox’s proportional hazards regression model. Sample size for each group was presented in the respective figure and table legends. A *p* < 0.05 was considered statistically significant.

## Results

### Flow Chart

**FIGURE 2:**
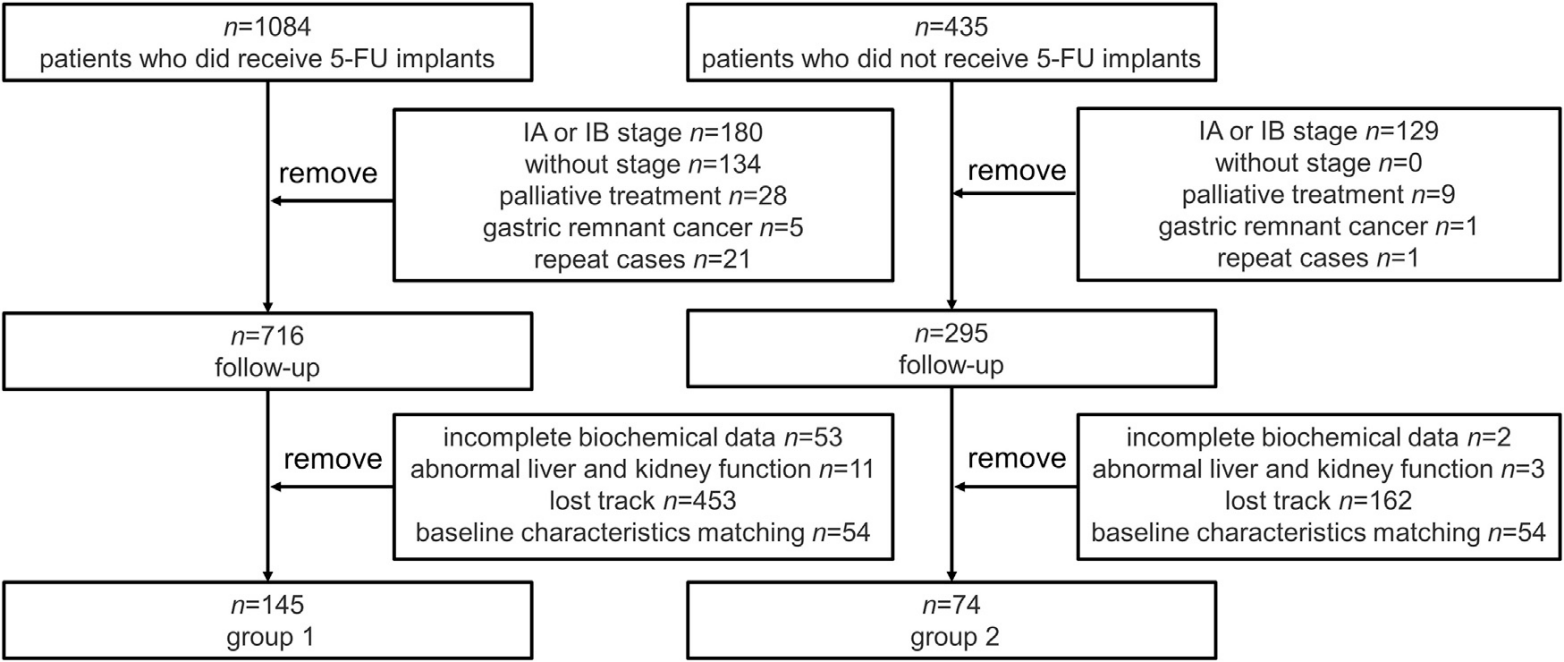
Flowchart of the selection of included patients. Repeat cases: The exactly same cases during the retrieval of patient data. 5-FU, 5-fluorouracil.

Data were collected from GC patients (*n* = 1,084) who received 5-FU implants during postoperative chemotherapy and patients who had not received 5-FU implants chemotherapy (*n* = 235) at the same time. According to the inclusion criteria, other clinical stages, palliative care, gastric remnant cancer and repeat cases were excluded. Then, there were 716 patients enrolled in follow-up in 5-FU group (group 1) and 295 in control group (group 2) respectively. After excluding incomplete data (53 in group 1 and 2 in group 2), loss of follow-up (453 in group 1 and 162 in group 2), and hepatic and renal failure (11 in group 1 and 3 in group 2), baseline matching was performed in the two groups. 54 patients were excluded in both group because of significant differences in gender, age, clinical stage, and surgical procedure during baseline matching. Finally, *n* = 145 individuals were enrolled in group 1, *n* = 74 individuals were enrolled in group 2. The flow of the included participants in the study is shown in Figure 2.

### Patients’ Characteristics

As shown in Table 1, no significant difference was found in age, sex, clinical stages, and operation method between the two groups (*p* > 0.05). Thus, the patients’ characteristics were relatively similar.

**TABLE 1:** Baseline clinical characteristics of the patients.

Parameter	5-FU (*n* = 145)	Control (*n* = 74)	*p* Value
Age (years)	60.26 ± 10.41	57.31 ± 10.86	0.052
Sex			0.762
Males	95	50	
Females	50	24	
AJCC stages^a^			0.898
Ⅱ	84	41	
Ⅲ	59	32	
Ⅳ	2	1	
Operation approach			0.625
Open operation	126	66	
Laparoscopic surgery	19	8	

^a^ Clinical tumor stage was assessed according to the eighth version of the American Joint Committee on *Cancer* (AJCC).

5-FU, 5-fluorouracil.

Data were expressed as MEAN ± SD.

### Postoperative Complications and Adverse Effects

The common complications and adverse effects occurred in gastric cancer patients after surgical operation are listed in Table 2 and Table 3. The two groups of patients from first day of post operation until the patients discharged from hospital safely were closely observed, and the time was about 8 days. The length of hospital stay after operation, time to first flatus and defecation were recorded and counted, no significant differences were found (*p* > 0.05). Moreover, there were no significant differences in pelvic infection, peritoneal infection, anastomotic leakage, incisional infection, ileus, and postoperative hemorrhage between the two groups (*p* > 0.05). Similarly, significant differences were not found in the incidence of fever, diarrhea, nausea, and vomiting among the two groups (*p* > 0.05). Compared to the control group, the biochemical parameters and the number of adverse reactions in the 5-FU implants group did not change significantly.

**TABLE 2:** Postoperative complications, length of hospital stay and adverse effects in patients.

Parameter	5-FU (*n* = 145)	Control (*n* = 74)	*p* Value
Hospital stay after operation (days)	8.55 ± 3.14	8.77 ± 3.84	0.670
Time to first flatus (days)	3.93 ± 1.68	3.79 ± 1.57	0.593
First defecation time (days)	4.81 ± 2.02	5.33 ± 1.89	0.072
Peritoneal infection	2	1	1.000
Pelvic effusion	3	1	1.000
Anastomotic leakage	0	0	-
Incisional infection	4	1	0.851
Ileus	2	3	0.443
Postoperative hemorrhage	3	1	1.000
Fever	11	8	0.432
Diarrhea	5	1	0.639
Nausea/vomiting	5	5	0.450

Data were expressed as MEAN ± SD.

5-FU, 5-fluorouracil.

**TABLE 3:** Postoperative complications and adverse effects in patients.

Parameter	5-FU (*n* = 145)				Control (*n* = 74)			
	Ⅰ	Ⅱ	Ⅲ	Ⅳ	Ⅰ	Ⅱ	Ⅲ	Ⅳ
Peritoneal effusion	1	1	0	0	0	0	1	0
Pelvic effusion	2	1	0	0	1	0	0	0
Fever	7	4	0	0	1	7	0	0
Diarrhea	1	4	0	0	0	0	1	0
Nausea/Vomiting	4	1	0	0	5	0	0	0
Anastomotic leakage	0	0	0	0	0	0	0	0
Pulmonary infection	0	2	0	0	1	6	0	0
Incisional infection	0	4	0	0	0	1	0	0
Ileus	1	0	1	0	0	3	0	0
Postoperative hemorrhage	1	2	0	0	0	1	0	0

Complications and adverse events were classified according to the National *Cancer* Institute common terminology criteria for adverse events (version 4.0) (Supplementary Table S1).

5-FU, 5-fluorouracil.

### Toxic Effects in the Two Groups

Toxic effects in both groups are shown in Table 4. When assessing hematologic toxicity, it was found that the level of RBC reduced significantly in the 5-FU implants group (*p* < 0.01) and the control group (*p* < 0.05) compared to baseline; and the levels of WBC (*p* < 0.01) increased in both groups after surgery. On the other hand, the liver functional index, including ALT (*p* < 0.01) and AST (*p* < 0.05) were elevated significantly after surgery. PLT and Cr did not change significantly after surgery in both groups. Postoperative RBC, WBC, PLT, AST, ALT and Cr in the 5-FU implants group were not significantly different from those in the control group. It could be suggested that 5-FU implants should not cause excessive toxicity.

**TABLE 4:** Hematologic toxic in patients.

Parameter	5-FU implants		Control	
	Preoperative	Postoperative	Preoperative	Postoperative
RBC (10^12^/L)	4.05 (0.67)	3.79 (0.57)**	4.00 (0.69)	3.74 (0.58)*
WBC (10^9^/L)	6.06 (1.88)	12.24 (5.70)**	6.32 (1.80)	12.34 (4.38)**
PLT (10^9^/L)	225.92 (72.66)	233.49 (101.04)	218.81 (75.28)	221.85 (89.85)
ALT (U/L)	21.26 (16.60)	45.38 (56.44)**	19.33 (14.63)	38.85 (62.91)**
AST (U/L)	24.22 (14.41)	44.11 (52.96)*	24.82 (21.86)	50.83 (157.86)*
Cr (μmol/L)	79.83 (16.25)	78.69 (21.26)	84.09 (14.72)	78.31 (17.35)

*Compared to preoperative, *p < 0.05, **p < 0.01, Data were expressed as MEAN (SD). Postoperative RBC, WBC, PLT, AST, ALT and Cr in the 5-FU implants group were not significantly different from those in the control group (*p* > 0.05).

5-FU, 5-fluorouracil; AST, aspartate aminotransferase; ALT, alanine aminotransferase; Cr, creatinine; PLT, platelet; RBC, red blood cell; WBC, white blood cell.

### Long-Term Results

The median follow-up time was 85 months (ranging from 60 to 116 months). 31 patients (21.38%) died of tumor recurrence in 5-FU implants group and 21 (28.38%) in control. 3 patients (4.05%) in control group were diagnosed metastases, the sites of metastasis appeared at the small intestine, left adrenal gland, and peritoneum.

**FIGURE 3:**
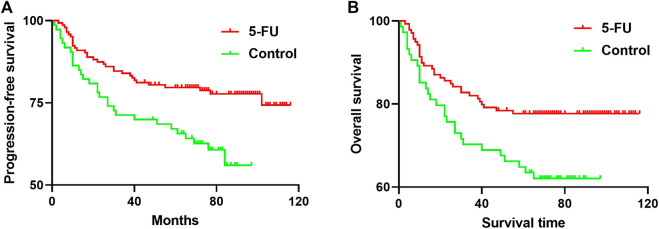
**(A)** Progression-free survival (PFS) curves (FU, red line; control, green line). The 5-year PFS was 79.71% in 5-FU implants group and 67.12% in control group (*p* = 0.0045) **(B)** Overall survival curves (FU, red line; control, green line). The group-specific 5-years overall survival (OS) rates were 77.68% in 5-FU implants group and 64.87% in control group. These differences were statistically significant (*p* = 0.0159). 5-FU, 5-fluorouracil.

Mean PFS was 95.44 months in 5-FU implants group and 68.88 months in control. The 5-year PFS was 79.71% in 5-FU implants group and 67.12% in control (*p* = 0.0045, Figure 3A). The group-specific 5-years OS rates were 77.68% in 5-FU implants group and 64.87% in control (*p* = 0.0159, Figure 3B).

### Prognostic Factors

The results of univariate analysis and the Cox model are shown in Table 5. On the basis of univariate analysis, we found that administration of 5-FU implants (*p* < 0.05), lymph node involvement (*p* < 0.01), and AJCC stage (*p* < 0.01) had a significant effect on the 5-years OS and PFS rate. Furthermore, the Cox model indicated that 5-FU implants can reduce the risk of 54.1% [95% CI: 0.303–0.967; *p* = 0.038] for OS and 52.7% [95% CI: 0.298–0.932; *p* = 0.028] for PFS. The death risk of patients with N3 GC was 6.685 [95% CI: 2.536–17.622; *p* = 0.000] times that of N0 GC. Since the AJCC stage did not have a significant difference in the prognosis of GC, it was excluded from the model. Therefore, 5-FU implants and lymph node involvement were important factors associated with prognosis of patients with GC.

**TABLE 5:** Univariate and multivariate survival analysis of overall survival (OS) and Progression-free survival (PFS) predictors.

Parameter	Univariate analysis			Multivariate analysis			Univariate analysis			Multivariate analysis		
	5-years OS (%)	Log rank χ^2^	*p* Value	HR	95% CI	*p* Value	5-years PFS (%)	Log rank χ^2^	*p* Value	HR	95% CI	*p* Value
Age (years)		0.003	0.956					0.020	0.887			
≥65	72.7						73.4					
<65	73.4						76.3					
Sex		0.573	0.449					1.488	0.223			
Males	75.0						77.7					
Females	69.9						71.1					
AJCC stages^a^		27.758	0.000			0.302		24.003	0.000			0.224
Ⅱ	85.5						86.3					
Ⅲ	57.0						61.6					
Ⅳ	33.3						33.3					
Depth of invasion		6.057	0.109					6.666	0.083			
T1	80.0						80.0					
T2	85.7						85.7					
T3	70.5						73.3					
T4	40.0						40.0					
Lymph node involvement		26.786	0.000					22.672	0.000			
N0	88.6			References			90.8			References		
N1	80.0			1.971	0.660–5.886	0.224	80.0			1.823	0.662–5.021	0.245
N2	72.2			2.773	0.947–8.120	0.063	75.2			2.480	0.916–6.714	0.074
N3	45.0			6.685	2.536–17.622	0.000	49.6			5.730	2.328–14.102	0.000
Adjuvant chemotherapy		5.814	0.016					8.072	0.004			
Non-5-FU implants	77.7			References			67.1			References		
5-FU implants	64.9			0.541	0.303–0.967	0.038	79.7			0.527	0.298–0.932	0.028
Operation approach		0.079	0.779					0.202	0.653			
Open operation	73.3						74.7					
Laparoscopic surgery	72.9						80.8					

^a^ Clinical tumor stage was assessed according to the eighth version of the American Joint Committee on *Cancer* (AJCC).

5-FU, 5-fluorouracil; CI, confidence interval; HR, hazard ratio. The bold indicates significant differences.

## Discussion

As the first drug used for the treatment of gastrointestinal malignant tumors, 5-FU has been widely recognized by clinicians (Chen et al., 2006; Wagner et al., 2017), which also provides an attractive choice when comes to the treatment of gastrointestinal tumors. Several studies have demonstrated that the therapeutic effect of infusional 5-FU in GC is considerable (Kang et al., 2014; Martínez-Lago et al., 2015). The 5-years OS rate of IB-IIIB GC patients treated with intravenous 5-FU chemotherapy was over 60% (Chang et al., 2002). However, another study have pointed out that treatment with 5-FU alone has little effect on survival rate (Cai et al., 2018). With furthering progress of the research, problems has emerged from traditional administration of 5-FU, which ranged from unstable blood concentration (Matsumoto et al., 2015) to high risk of toxicity, including cardiotoxicity (Peng et al., 2018), neutropenia (Zhang et al., 2018), thrombocytopenia and anemia (De Vita et al., 2007). These problems bring out some negative impacts on the treatment and prognosis of patients.

Combined application of chemotherapeutic drugs is a common approach in our department that has been testified improving their clinical therapeutic effect, and widely used in postoperative chemotherapy. Regime of 5-FU plus cisplatin is recommended the first-line chemotherapy for GC in Asia (Qu et al., 2015). A study showed long-term infusion of low-dose 5-FU plus cisplatin in advanced GC patients, the 1-year survival rate was 36.2%. Drug toxicity during treatment could be tolerated (Kim et al., 2000). The regime also showed effective for recurrent GC (the efficacy rate was 34.6%), and adverse effects were controllable (Kanetaka et al., 2012). However, another study found that the combined application of 5-FU and cisplatin as adjuvant chemotherapy for postoperative GC patients would insignificantly increase five-year and seven-year OS rates, compared with the control group (surgery-only) (Bouché et al., 2005). S-1, an oral fluoropyrimidine, combined with oxaliplatin (SOX) could increase the three-year DFS rate to 74.3% (S. Park et al., 2020) and overall response rate to 40% (Kim et al., 2012). Bang et al. indicated that three-year disease-free survival rate of oxaliplatin and capecitabine as adjuvant chemotherapy was significantly higher than that of the D2 gastrectomy-only group (74 vs. 59%). However, the incidence of serious adverse reactions obviously increased due to the cumulative toxic effect of oxaliplatin (Bang et al., 2012).

Combination therapy is also used for preoperative chemotherapy. FLOT (fluorouracil, leucovorin, oxaliplatin, and docetaxel) could improve survival rate of patients with locally advanced, resectable GC in the perioperative period. The five-year OS rate of FOLT group reached 45%, which was significantly higher than the 36% of the control group (fluorouracil or capecitabine plus cisplatin and epirubicin). The incidence of serious adverse reactions in both groups was 27% (Al-Batran et al., 2019).

In the prevention of peritoneal metastases, intraperitoneal chemotherapy has the unique advantage of acting directly on the cancer cells in the peritoneal cavity at a higher concentration of drug. More importantly, intraperitoneal chemotherapy is safe and reliable.(Witkamp et al., 2001; Kobayashi and Kodera, 2017). Paclitaxel intraperitoneal chemotherapy combined with systemic chemotherapy (S-1 plus intravenous paclitaxel) significantly improved 3-year OS rate compared to systemic chemotherapy (intravenous cisplatin plus S-1) alone (21.9 vs. 6.0%) (Ishigami et al., 2018). It had been revealed from multiple studies that HIPEC could increase the OS rate of patients and reduce the incidence of peritoneal metastasis with advanced GC (van der Speeten et al., 2009; Brenkman et al., 2019). Nevertheless, controversy still exists in the prognosis of GC (Scaringi et al., 2008; Chia et al., 2016).

Currently, the application of sustained-release preparations in cancer chemotherapy is becoming more and more extensive. The development and evaluation of sustained-release preparations of chemotherapeutic drugs have also become an important task for cancer treatment. Some studies in recent years have confirmed the distinctive advantages of sustained-release preparations in cancer treatment. Ling et al. evaluated the pharmacokinetics and biodistribution of 5-FU-loaded PLGA implants in a nude mouse model with colon cancer. The result showed that the concentration, C_max_ and area under curve (AUC) of 5-FU in the peritoneal fluid were higher than that in plasma, which significantly increased the accumulation time of the drug in the tumor tissue, improved the anti-tumor activity and reduced the adverse reaction (Li et al., 2018). In addition, the C_max_ around the tumor in the 5-FU implant group was approximately 4–5 times that of intraperitoneal injection. Compared with systemic administration, 5-FU implants had higher local drug concentration in the tumor-bearing mice (Chen et al., 2014). An *in vivo* study in cancer patients comprehensively evaluated the pharmacokinetic characteristics of 5-fluorouracil implants. Within 24 h after 5-FU implantation, a high drug concentration of 0.5–0.6 mg/L was formed in the implanted area. After the peak concentration, a relatively stable release rate was formed. The half-life of 5-FU in sustained-release chemotherapy is (11,364.0 ± 6.8) minutes, which was 1,045.23 times of systemic chemotherapy. The effective concentration of the drug sustained release time is 360–500 h (Li et al., 2007). Some researchers combined 5-FU implants with systemic chemotherapy to treat patients with pancreatic cancer and found that it could improve the survival of patients compared with systemic chemotherapy alone (Li et al., 2016). Therefore, 5-FU implants were more effective and safer than direct injection.

Hang et al. reported that the 5-year survival rate of colorectal cancer patients who received 5-FU implants for chemotherapy was 56.12%, which was significantly better than the control group*.* And the 5-year disease-free survival rate was 48.98% in the 5-FU implants group and 34.62% in control group (Yuan et al., 2015). In addition, the adverse effects of advanced GC patients administrated 5-FU implants at the end of surgery did not increase, the 3-year survival rate was 64.3%, significantly higher than control group (Liu et al., 2014). The incidence of complications between the two groups was undifferentiated. In summary, radical surgery plus 5-FU implants did not increase the incidence of complication nor cause more adverse effects to patients (Liu et al., 2014; Yuan et al., 2015). Our results were similar to previous studies, which showed that 5-FU implants improved the long-term survival rate (77.68 vs. 64.87%) and PFS rate (79.71 vs. 67.12%) of patients with GC. According to follow-up results, 5-FU implants intraperitoneal chemotherapy can significantly improve the treatment effect and prognosis of patients with GC. In addition, according to the results of Cox’s Proportional Hazards Regression Model, 5- FU implants and lymph node involvement were the key factors affecting the quality of life. However, previous studies showed that multiple factors, such as age, TNM (tumor-node-metastasis) and clinical stage, were associated with the risk of death in patients with GC (Zhang et al., 2018; Orman and Cayci, 2019), but we did not conclude that other factors, such as clinical stage, were independent predictors. It may be due to individual patient differences and sample size limitations.

There are still several limitations in our study, such as the certain cardiotoxicity of 5-FU is not involved in this study, we need further studies to verify whether 5-FU implants could reduce cardiotoxicity. Due to the small number of patients eventually included in this study, the drug evaluation has not been verified on a large scale. During the period of study process, a large number of patients were lost to follow-up, resulting in a limited sample size. The clinical efficacy of 5-FU implants drugs requires a larger clinical research to be accurately evaluated. According to the data of peritoneal metastasis in patients with stage Ⅳ gastric cancer in our department (unpublished data), peritoneal metastasis is still an important factor affecting the prognosis of GC patients. However, perhaps due to the low rate of peritoneal metastasis in stage Ⅱ and Ⅲ patients (Thomassen et al., 2014), no significant difference was observed between the two groups in our study. The occurrence of peritoneal metastasis needs to be observed in a large population sample, and cannot rely solely on the institution data. The prevention and treatment of peritoneal metastasis also need new research to further explore.

## Conclusion

The application of 5-FU implants in GC surgery significantly improves the five-year survival rate of patients. Moreover, it has good safety and provides a basis for the treatment and prognosis of gastric cancer. Therefore, the 5-FU implants have proven to be an effective treatment option for gastric cancer.

## Data Availability

The raw data supporting the conclusions of this article will be made available by the authors, without undue reservation.
