# Supplementary material for: Retrospective Cohort Study of Intraoperative Administration of Sustained-Release 5-Fluorouracil Implants in Advanced Gastric Cancer Patients
Source: Front Pharmacol. 2021 Apr 13;12:659258. doi: 10.3389/fphar.2021.659258 (PMC8076801; doi:10.3389/fphar.2021.659258)
Supplement: Supplementary file 1 [file table1.docx]

Supplementary table 1 Grading criteria for postoperative complications and adverse events

| Parameter | Ⅰ | Ⅱ | Ⅲ | Ⅳ |
| --- | --- | --- | --- | --- |
| Pelvic effusion | Asymptomatic; clinical or diagnostic observations only; intervention not indicated | Symptomatic; medical intervention indicated | Severe symptoms; invasive intervention indicated | Life-threatening consequences; urgent operative intervention indicated |
| Peritoneal effusion | Asymptomatic; clinical or diagnostic observations only; intervention not indicated | Symptomatic; medical intervention indicated | Severe symptoms; invasive intervention indicated | Life-threatening consequences; urgent operative intervention indicated |
| Fever | 38.0 - 39.0 degrees C (100.4 - 102.2 degrees F) | >39.0 - 40.0 degrees C (102.3 - 104.0 degrees F) | >40.0 degrees C (>104.0 degrees F) for <=24 hrs | >40.0 degrees C (>104.0 degrees F) for >24 hrs |
| Diarrhea | Increase of <4 stools per day over baseline; mild increase in ostomy output compared to baseline | Increase of 4 - 6 stools per day over baseline; moderate increase in ostomy output compared to baseline | Increase of >=7 stools per day over baseline; incontinence; hospitalization indicated; severe increase in ostomy output compared to baseline; limiting self care ADL | Life-threatening consequences; urgent intervention indicated |
| Nausea | Loss of appetite without alteration in eating habits | Oral intake decreased without significant weight loss, dehydration or malnutrition | Inadequate oral caloric or fluid intake; tube feeding, TPN, or hospitalization indicated | - |
| Vomiting | 1 - 2 episodes (separated by 5 minutes) in 24 hrs | 3 - 5 episodes (separated by 5 minutes) in 24 hrs | >=6 episodes (separated by 5 minutes) in 24 hrs; tube feeding, TPN or hospitalization indicated | Life-threatening consequences; urgent intervention indicated |
| Anastomotic leakage | Asymptomatic diagnostic observations only; intervention not indicated | Symptomatic; medical intervention indicated | Severe symptoms; radiologic, endoscopic or elective operative intervention indicated | Life-threatening consequences; urgent operative intervention indicated |
| Pulmonary infection | - | Moderate symptoms; oral intervention indicated (e.g., antibiotic, antifungal, antiviral) | IV antibiotic, antifungal, or antiviral intervention indicated; radiologic or operative intervention indicated | Life-threatening consequences; urgent intervention indicated |
| Incision infection | - | Localized; local intervention indicated (e.g., topical antibiotic, antifungal, or antiviral) | IV antibiotic, antifungal, or antiviral intervention indicated; radiologic or operative intervention indicated | Life-threatening consequences; urgent intervention indicated |
| Ileus | - | Symptomatic; altered GI function; bowel rest indicated | Severely altered GI function; TPN indicated | Life-threatening consequences; urgent intervention indicated |
| Postoperative hemorrhage | Minimal bleeding identified on clinical exam; intervention not  indicated | Moderate bleeding; radiologic, endoscopic, or operative intervention indicated | Transfusion indicated of >=2 units (10 cc/kg for pediatrics) pRBCs beyond protocol specification; urgent radiologic, endoscopic, or operative intervention indicated | Life-threatening consequences; urgent intervention indicated |

ADL: activities of daily living; GI: gastrointestinal; IV: intravenous; pRBC: packed red blood cells; TPN: total parenteral nutrition
